# Association Between Anxiety and Diet Quality Among Polish Adults: A Cross-Sectional Study

**DOI:** 10.3390/nu17223508

**Published:** 2025-11-10

**Authors:** Paulina Sławińska, Ewa Piotrowska, Karolina Rak, Ewa Raczkowska

**Affiliations:** Department of Human Nutrition, Faculty of Biotechnology and Food Science, Wrocław University of Environmental and Life Sciences, 37 Chełmońskiego Street, 51-630 Wrocław, Poland; 121419@student.upwr.edu.pl (P.S.); ewa.piotrowska@upwr.edu.pl (E.P.); karolina.rak@upwr.edu.pl (K.R.)

**Keywords:** anxiety, anxiety disorders, diet quality, eating habits, food choices

## Abstract

Background/Objectives: Anxiety can influence dietary choices and habits, but dietary choices and habits can also contribute to the intensification of anxiety symptoms. The study aimed to test the hypothesis that a higher level of anxiety predicts poorer diet quality among adults in Poland. Methods: A cross-sectional survey was conducted among 1841 individuals aged 18 years and older across Poland. A self-developed survey drew upon the KomPAN questionnaire, the Healthy Eating Plate with its accompanying infographic and the Penn State Worry Questionnaire (PSWQ). Multivariable logistic regression analysis was used to assess the relationship between anxiety level and gender, age, nutritional status, and other sociodemographic factors. The same approach was applied to evaluate the relationship between diet quality and the aforementioned variables. In addition, hierarchical clustering of variables was performed using Ward’s method. Results: Nearly half of the respondents presented a high level of anxiety (48.29%), while most reported a low-quality diet (64.58%). Participants aged 18–22 years were significantly more likely to exhibit both high anxiety levels (aOR = 1.614; 95% CI: 1.327–1.964; *p* < 0.001) and low diet quality (aOR = 1.810; 95% CI: 1.482–2.211; *p* < 0.001) compared to older groups. The findings support the hypothesis that higher anxiety levels are linked to poorer diet quality, particularly among young adults. Conclusions: Higher levels of anxiety were shown to be significantly associated with poorer diet quality, with the strongest effects observed in the youngest age group. These results highlight the need for integrated psychological and nutritional interventions targeting this group. Further longitudinal studies are warranted to clarify the directionality of the observed associations.

## 1. Introduction

Until recently, terms like “anxiety” or “anxiety disorders” were rarely used in public discourse. Today, they represent a growing public health concern.

Anxiety is a state similar to fear, although there are some differences between them. Fear is a direct response of the body to an existing threat, while anxiety involves increased attention, heightened vigilance, and avoidance of potential danger [1]. Anxiety disorders are conditions characterized by persistent and disproportionate fear and worry. These disorders are also associated with specific behaviors and symptoms that hinder daily functioning, such as physical symptoms (e.g., shortness of breath, nausea, heart palpitations, fatigue, abdominal discomfort) [1,2].

Anxiety disorders are among the most common mental health conditions worldwide. They occur more frequently in women than in men. In 2019, they affected 301 million people worldwide [3]. Their onset most often occurs during childhood, adolescence, or early adulthood [1,3]. The underlying causes of anxiety disorders are believed to include negative emotions linked to childhood events, as well as genetic predispositions to structural and functional changes in the central nervous system [4,5]. According to current standards, the first-line treatment for anxiety disorders is psychotherapy, which may be combined with pharmacotherapy. The most commonly used medications in treatment are selective serotonin reuptake inhibitors (SSRIs) and serotonin-norepinephrine reuptake inhibitors (SNRIs) [6]. Dietary therapy and lifestyle modifications may serve as beneficial adjuncts to standard treatments. As demonstrated by a review and meta-analysis of 96 studies, such interventions led to improvements in the well-being of individuals experiencing depression, anxiety, or stress [7].

One of the theories that attempts to explain the phenomenon of emotional eating is the psychosomatic theory, which suggests that negative emotions contribute to increased food intake. In such cases, eating serves to alleviate negative emotions; but overeating itself arises from these emotions [8]. The causes of emotional eating may include, among others: the desire to escape negatively charged self-awareness, confusing hunger or thirst with intense emotional experiences, and the suppression of emotions [9]. For example, during the COVID-19 pandemic period, many people consumed more food and alcohol than before in an attempt to cope with the emotions they were experiencing at the time [10]. Negative emotions can lead to a lack of interest in food [11], a decreased desire to eat preferred foods [12], or, conversely, may result in increased food intake, especially of sweet products [13]. Increased consumption of chocolate and confectionery products may be associated with a higher risk of depression and anxiety symptoms [14]. One analysis indicates a link between higher levels of anxiety and a high sugar and refined carbohydrate intake, a high fat-diet, insufficient dietary protein and tryptophan intake, as well as unhealthy eating habits [15]. The gut–brain axis also plays a key role. Gut microbiota can synthesize neurotransmitters, including dopamine, serotonin, and γ-aminobutyric acid (GABA) [16]. Abnormalities in the concentrations of these neurotransmitters have been associated with the manifestation of anxiety symptoms [17]. Furthermore, gut dysbiosis may increase intestinal permeability, allowing harmful metabolites to enter the bloodstream and induce inflammatory responses. Consequently, this may affect the nervous system and its ability to regulate anxiety responses [17]. A study examining the impact of diet quality on the microbiome, and thereby on anxiety levels, presented findings showing a negative influence of the Western diet—rich in processed foods—on higher anxiety levels. However, the exact mechanism remains unclear. The adverse effects of the Western diet may stem from the proinflammatory properties of processed foods, which affect gut microbiota composition, as well as from high sugar intake, which may increase intestinal permeability [18].

A stressful event can suppress the desire to eat through the action of corticotropin-releasing hormone (CRH). However, several hours or even days later, glucocorticoid levels rise, which not only contribute to the accumulation of visceral fat but also stimulate appetite by acting on the hypothalamus. Excessive glucocorticoid levels may occur in chronic stress and mood disorders, potentially leading to excessive intake of high-calorie foods, although in some individuals the opposite effect—reduced food intake—may be observed. This is likely related to ghrelin levels: individuals who eat more food under emotional influence have lower ghrelin levels compared to those who consume the same amount or less [19].

Although research on the relationship between emotions and food is extensive and growing, the majority of studies focus on stress [20] or sadness [13]. Anxiety is taken into account to a much lesser extent, despite being one of the most common mental health problems worldwide. Many previous analyses were based on cross-sectional studies with limited sample sizes or did not include important moderating variables, such as gender, age, or level of education [21,22]. For these reasons, there is a clear need for further, more detailed research—both nutritional and psychological—to determine whether dietary patterns influence anxiety levels, or whether anxiety itself defines diet quality and shapes the choice of specific foods. The present study sought to address this gap by analyzing the relationships between anxiety levels and the frequency and type of foods consumed, eating habits, and the tendency toward emotional eating in a representative group of respondents. In view of the above, it was assumed that the level of anxiety experienced in everyday life affects the quality of the diet and thus determines the eating habits and food choices of respondents.

## 2. Materials and Methods

### 2.1. Study Design and Sample

The research was conducted from April 2024 to March 2025. The data collection method used was the snowball sampling method. In this study, the research group consisted of women and men aged 18 and over. The survey method was used to collect data for this study. Google Forms was used to design the questionnaire. Responses were collected by sharing the questionnaire online. The questionnaire could be completed by any willing participants who were legal age and fluent in Polish. The exclusion criterion for study was being under the age of 18. A total of 1841 people participated in the study, of whom 91.96% were women and 8.04% were men. According to data from the Central Statistical Office of Poland, the country’s population at the end of 2024 was 37.489 million. The adult population (18+) is estimated at approximately 30.7 million people [23]. The sample size therefore ensures a high level of result precision—at a 95% confidence level, the maximum margin of error does not exceed ±2.3%. This value is considered acceptable in public opinion and social research [24,25]. The assessment of nutritional status was performed using the Body Mass Index (BMI), calculated based on the self-reported body weight and height of the respondents, and classified as follows:

underweight <18.50 kg/m^2^,
normal weight <18.50–24.99 kg/m^2^>,
overweight <25.00–29.99 kg/m^2^>,
obesity class I <30.00–34.99 kg/m^2^>,
obesity class II <35.00–39.99 kg/m^2^>,
obesity class III >39.99 kg/m^2^ [26].

The research was approved by the Research Ethics Committee of Wrocław University of Life Sciences (no. 14/2023 of 29 June 2023). The study was conducted in accordance with the guidelines of the Declaration of Helsinki [27].

### 2.2. Questionnaire

The survey questionnaire consisted of four parts and included a total of 49 questions. The first part contained 11 questions and concerned general eating behaviors, including, among others, meal regularity and snacking between meals. It was developed based on selected questions from the KomPAN questionnaire [28]. The second part, comprising 9 questions, was created based on the Healthy Eating Plate and its accompanying infographic titled “Three Steps to Health” [29]. This section aimed to assess the dietary quality of the respondents. The third part, consisting of 20 questions, was developed using the Penn State Worry Questionnaire (PSWQ) [30] and original questions. It focused on measuring the level of anxiety experienced by the participants, its causes, its impact on appetite, and the eating behaviors and choices made during periods of anxiety. The final part, which included 9 questions, was the sociodemographic section. The questions in this section concerned gender, age, height and weight, place of residence, education, employment status, marital status, and the number of people in the household. The questionnaire was not validated, as it was based on previously validated tools.

#### 2.2.1. Level of Anxiety

To assess the level of anxiety, an anxiety level index was created based on the Penn State Worry Questionnaire (PSWQ) [30]. Participants were asked to indicate on a 5-point scale the extent to which they identified with each statement. The highest possible score was 80, and the lowest was 16, with higher scores indicating a greater tendency to worry and experience anxiety. Three levels of anxiety were distinguished: low (16–39 points), moderate (40–59 points) and high (60–80 points).

#### 2.2.2. Diet Quality

To assess diet quality, an index of healthy eating and physical activity recommendations called “The Road to Health” was developed based on the Healthy Eating Plate and the accompanying infographic “Three Steps to Health” [29], as well as on the official dietary guidelines of the National Institute of Public Health. The tool consisted of eleven items encompassing key elements of a healthy lifestyle: (1) regularity of main meals, (2) snacking between meals, (3) physical activity, and the frequency of consumption of (4) fish, (5) milk and dairy products, (6) fats, (7) sweets and sweetened beverages, (8) vegetables and fruits, (9) meat and meat products, (10) cereal products, and (11) salt. For each item, participants selected one of three response options corresponding to the “Three Steps to Health” model. Each step reflected a progressively higher level of adherence to recommendations and was scored as follows: 0 points (lack of adherence), 1 point (low adherence), 2 points (moderate adherence), or 3 points (full adherence). Intermediate options (e.g., partial adherence) were allowed., with increments of 0.5 points, resulting in a possible total score ranging from 0 to 28.5 points. The overall index score was obtained by summing the points from all items. A higher total score indicated better compliance with healthy dietary and lifestyle habits. Based on the theoretical score distribution an expert consultation, three levels of diet quality were defined:

- low diet quality: 0.0–9.4 points,
- moderate diet quality: 9.5–19.0 points,
- high diet quality: 19.1–28.5 points.

This classification was intended to reflect gradual progress in achieving healthy eating habits The “Road to Health” tool was developed specifically for this study to capture the degree of adherence to healthy eating recommendations within the Polish population. As the index has not undergone formal psychometric validation, it should be regarded as an exploratory measure. The classification thresholds for diet quality (low, moderate, high) were established based on theoretical distribution and expert consensus rather than empirical validation. Future research should aim to confirm the validity and reliability of this instrument in independent samples.

### 2.3. Statistical Analysis

Qualitative variables were presented as percentages (%). The chi-square test was used to verify differences between these variables. Multivariable logistic regression (enter method) was applied to assess the independent associations between anxiety level/diet quality and demographic, anthropometric, and socioeconomic factors. All predictors were entered into the model simultaneously to obtain adjusted odds ratios (aOR) controlling for potential confounders including age, gender, BMI category, place of residence, educational level, occupational status, marital status, and household size. Adjusted odds ratios (aOR) were calculated with a 95% confidence level.

To analyze the structure of the variables, hierarchical cluster analysis was conducted using Ward’s method and Euclidean distance as the measure of similarity. The aim of this analysis was to identify groups of variables with similar occurrence profiles. The results were presented as a dendrogram, which visualizes the process of grouping variables into successive clusters based on their degree of similarity. A higher linkage distance indicates a lower similarity between the merged groups. A *p*-value below 0.05 was considered statistically significant for all tests. Statistical analysis was performed using the STATISTICA software package (version 13.3, PL; StatSoft Inc., Tulsa, OK, USA; StatSoft, Kraków, Poland).

## 3. Results

### 3.1. Characteristics of the Study Sample

Table 1 presents the socio-demographic characteristics of the study group. Women accounted for 91.96% of respondents, while men made up 8.04%. Individuals aged 18–22 years represented nearly two thirds of all respondents. Almost 60% of respondents had normal body weight, while nearly one third were overweight. The largest proportion of respondents lived in cities with over 100,000 inhabitants. The majority of respondents had a secondary education (63.12%), while nearly 30% had higher education. Almost 70% of respondents were students. A slightly larger proportion were single. Nearly 50% lived in households of four and more.

### 3.2. The Relationship Between Anxiety Levels and Selected Sociodemographic Characteristics

Table 2 summarizes the statistics regarding anxiety levels among the surveyed respondents. Nearly half of the participants exhibited a high level of anxiety—48.29%. The second-largest group consisted of those with a moderate level of anxiety—38.78%. The smallest group was individuals with a low level of anxiety—12.93%. Considering gender, women had nearly three times higher odds of presenting high anxiety levels compared to men (aOR = 2.926; 95% CI: 1.999–4.282). Age was also an important determinant: participants aged 18–22 years were at significantly higher risk of high anxiety (aOR = 1.614; 95% CI: 1.327–1.964; *p* < 0.001) compared with the reference group (26–50 years), while respondents aged >50 years had the lowest odds. Underweight increased the risk of anxiety symptoms (aOR = 1.505; 95% CI: 1.128–2.008). Individuals with underweight were almost 1.5 times more likely to present high anxiety symptoms compared to those with obesity. Those with higher education had a lower risk of severe anxiety (aOR = 0.731; 95% CI: 0.597–0.894). Among occupational groups, students were at the greatest risk for high anxiety (aOR = 1.621; 95% CI: 1.330–1.975), pensioners (aOR = 0.327; 95% CI: 0.160–0.669) had a lower risk (Table 3).

### 3.3. The Relationship Between Diet Quality and Selected Sociodemographic Characteristics

Table 2 summarizes the statistics regarding diet quality among the surveyed respondents. Almost two-thirds of respondents had a low-quality diet—64.58%. The group with a moderate-quality diet accounted for 34.66%, while the smallest group consisted of individuals with a high-quality diet—0.76%.

The factor that most increased the risk of low quality was being in the 18–22 age group (aOR = 1.810; 95% CI: 1.482–2.211). However, the risk decreased with age. Participants aged 18–22 years showed nearly twice the odds of low diet quality relative to those aged >50 years. Both underweight (aOR = 1.663; 95% CI: 1.204–2.298) and overweight (aOR = 1.246; 95% CI: 1.011–1.536) were associated with low diet quality. Residents of rural areas were more likely to have a low-quality diet (aOR = 1.466; 95% CI: 1.189–1.807). Higher education correlated with a lower risk of poor dietary habits (aOR = 0.513; 95% CI: 0.417–0.630). Students (aOR = 1.333; 95% CI: 1.089–1.630) and unemployed individuals (aOR = 1.556, 95% CI: 1.013–2.389) more often had low-quality diets compared to pensioners (aOR = 0.540; 95% CI: 0.293–0.997). Those living alone (aOR = 0.698; 95% CI: 0.511–0.953) or in two-person households (aOR = 0.700; 95% CI: 0.559–0.877) were less likely to experience reduced diet quality (Table 4).

### 3.4. Segmentation of Respondents Based on Anxiety Level, Diet Quality, and Sociodemographic Characteristics—Ward’s Classification

**Figure 1:**
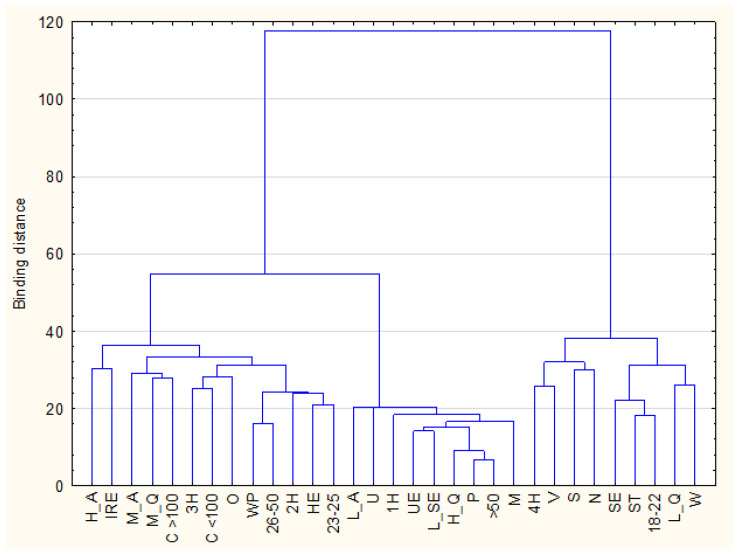
The hierarchical classification of variables describing high anxiety level, low diet quality, gender, age, BMI index, and socio-economic factors. W—women; M—men; 18–22—age in years; 23–25—age in years; 26–50—age in years; >50—age in years; U—underweight; N—normal weight; O—overweight; V—village; C < 100—city with up to 100,000 inhabitants; C >100—city with 100,000 and over inhabitants; L_SE—education lower than secondary; SE—secondary education; HE—higher education; ST—students; UE—unemployed; WP—people working professionally; P—pensioners; S—single; IRE—in a relationship; 1H—household of one; 2H—household of two; 3H—household of three; 4H—household of four and more; L_Q—low diet quality; M_Q—medium diet quality; H_Q—high diet quality; L_A—low anxiety level; M_A—medium anxiety level; H_A—high anxiety level.

Using Ward’s hierarchical clustering method, three variable clusters were identified (Figure 1). Hierarchical cluster analysis was used to identify subgroups distinguished by diet quality and anxiety levels. Ward’s method and Euclidean distance were applied to maximize within-cluster homogeneity and between-cluster differences. The number of clusters was selected based on the largest increase in linkage distance observed in the dendrogram, indicating the most meaningful segmentation of the data. The three resulting clusters reflected distinct behavioral and demographic profiles, offering a complementary, pattern-based perspective on the anxiety–diet relationship.

The first cluster included individuals with a low-quality diet, characterized by traits such as: female gender, age 18–22, student status, secondary education, normal body weight, being single, living in rural areas, and residing in households with four or more members. This pattern may reflect lifestyle instability, academic pressures, and limited autonomy in food choices, suggesting a population in need of targeted nutritional and psychological support. The second cluster consisted of individuals with low anxiety levels, a high-quality diet, as well as being unemployed or retired, having less than secondary education, being over 50 years old, living alone, and being male, which may reflect greater life stability, established health habits, and accumulated self-regulatory capacity. The third cluster included individuals with high and moderate anxiety levels and a moderate-quality diet, aged 23–50, with higher education, employed, overweight, in a relationship, living in cities with populations both above and below 100,000, and residing in two- or three—person households, possibly reflecting accumulated occupational and family responsibilities. As this clustering approach is exploratory and descriptive in nature, these interpretations should be considered hypothesis-generating rather than causal explanations.

To sum up, participants with high anxiety levels more frequently demonstrated poorer diet quality, and the adjusted odds ratios suggested a small to moderate effect size. The most pronounced differences were observed among younger individuals (18–22 years) and those with underweight or lower education levels.

## 4. Discussion

The aim of this study was to determine the level of anxiety experienced by respondents in their daily lives and whether anxiety is associated with respondents’ eating behaviors and food choices, and how these relationships may manifest in different groups. It was assumed that strong emotions, in this case anxiety, may influence eating habits, the types of foods chosen, the frequency of meals, and the quantity of food consumed, but also that dietary choices themselves may intensify the experience of anxiety.

Three levels of anxiety were distinguished: low, moderate, and high. The largest proportion of respondents exhibited a high level of anxiety. This may result from global issues such as terrorism, unemployment, economic crises including hunger, environmental and food contamination, civilization—related diseases—particularly obesity, type 2 diabetes, hypertension, cancer, emerging pathogenic viruses, or nuclear threats [31,32]. In the present study, women exhibited higher levels of anxiety compared to men. In multivariable logistic regression models, several sociodemographic factors remained significant predictors after adjusting for other covariates. Women demonstrated nearly a three-fold higher likelihood of experiencing high anxiety levels compared to men, even after controlling for age, BMI, education, and socioeconomic indicators. This indicates that gender remained an independent determinant of anxiety in the present sample. The same observation was reported by the authors of a study on international associations between gender and mental disorders. That study included 72,933 adults from Africa, North America, South America, Europe, Asia, the Middle East, and the Pacific. Women, compared to men, more frequently suffered from anxiety and mood disorders, whereas men more often experienced substance-related disorders [33]. Findings from Nepal also confirmed that women were more likely to experience anxiety (aOR = 2.18) [34]. In the present study, aOR = 2.926.

Younger individuals were more likely to experience anxiety than older participants. This trend was also reflected in the association between anxiety and occupational status. A 25-year longitudinal study reported that anxiety levels increased across all age groups except the oldest cohort. The largest increase was recorded in the 16–23 age group [35]. This may indicate that young adults live with increasing levels of anxiety, potentially due to concerns about the future, particularly climate change or armed conflicts [36,37], but also because of excessive screen time on smartphones, which may become addictive [38].

Considering education level, the present study showed that the higher the level of education, the lower the risk of experiencing anxiety. Murcia et al. [39] examined educational inequalities in depression and anxiety among 11,777 individuals, including 8072 employed participants. Their findings in the overall sample indicated a decline in the prevalence of anxiety disorders with higher levels of education. Comparing working and non-working participants, a clearly higher level of anxiety was observed among those who were unemployed. Among employed individuals, anxiety risk was lower; however, this relationship was not statistically significant in unemployed participants.

The present study further showed that underweight was associated with a higher occurrence of anxiety. This finding aligns with results by DeJesus et al. [40], who observed higher anxiety levels in individuals who were underweight or overweight compared with those of normal body weight. In contrast, our findings did not show a significant association between overweight and anxiety. However, de Wit et al. [41] reported that excessive body weight increased the risk of mood and anxiety disorders, although underweight status did not show a significant effect in their analysis.

Three levels of diet quality were distinguished: low, moderate, and high. The largest proportion of respondents reported a low-quality diet. In a review of 1541 scientific reports [15], an association was observed between lower anxiety levels and, among other factors, higher consumption of vegetables, fruits, omega-3 fatty acids, healthy eating habits, and regular breakfast consumption. The analysis also revealed a link between higher anxiety levels and high intake of sugar and refined carbohydrates, a high-fat diet, insufficient dietary protein and tryptophan intake, as well as unhealthy eating habits. In this study, most respondents reported high anxiety levels. This may reflect the impact of a low-quality diet on anxiety symptoms. Conversely, anxiety may promote unhealthy eating patterns, leading to reduced diet quality. It is also important to consider potential mechanisms that may explain the observed associations between anxiety and diet quality. One such mechanism involves activation of the hypothalamic–pituitary–adrenal (HPA) axis and the resulting increase in cortisol levels, which may enhance appetite for energy-dense, sweet, and high-fat foods. Evidence indicates that individuals with greater cortisol reactivity tend to increase caloric intake under stress and preferentially select highly palatable foods [42,43].

Considering age, in the present study, being 18–22 years old increased the risk of having a low-quality diet, whereas in older individuals this risk was lower. A study on the diet quality of German adolescents showed that their intake of vegetables and fruits, as well as milk and dairy products, was below recommended levels, while the intake of non-recommended foods such as sweets was 1.5 times higher than the recommended amount [44]. In a study by Gajda et al. [45] on dietary diversity and quality among older adults, it was found that nearly three-quarters of respondents had a moderately healthy diet. These findings are consistent with the present results. Underweight and overweight individuals were more likely to have a low-quality diet. Ford et al. [46] showed that individuals with a BMI below 18.5 kg/m^2^ had a lower-quality diet compared to those with normal body weight. That study focused on older adults, whereas the present study mostly included younger participants, suggesting that the relationship between body weight and diet quality may not be dependent on age. Gómez et al. [47] demonstrated that low diet quality was associated with low socioeconomic status. In the present study, such an indicator was not assessed, but it can be assumed that students and unemployed individuals have a lower socioeconomic status compared to employed individuals and therefore are at a higher risk of having a lower-quality diet. A similar relationship can be inferred regarding rural residents, who also showed a higher likelihood of a low-quality diet; this may likewise be related to socioeconomic status.

To expand upon the above observations, the present study also employed multivariate analysis. Accordingly, three sets of variables (clusters) were identified to describe anxiety level and diet quality. Given that no direct indicators of socioeconomic status (SES), income, or social support were collected, the interpretations of the three clusters should be regarded as exploratory hypotheses rather than confirmed mechanisms. Accordingly, the discussion below adopts a cautious, data-driven approach, supported by recent scientific evidence.

The first cluster comprised individuals characterized by a low-quality diet, mostly women aged 18–22, students with secondary education, of normal body weight, single, living in rural areas, and belonging to households with four or more members. As low SES is a known determinant of poorer dietary patterns, this combination of features could tentatively reflect a group at risk of lower socioeconomic resources. However, because our data did not directly measure SES, this interpretation remains hypothetical and should be treated cautiously. Several studies indicate that younger adults, especially students transitioning from their parental homes to university life, experience a decline in diet quality due to changes in food environment, cooking habits, and financial autonomy. Moreover, research among college students consistently links lower dietary quality with increased symptoms of anxiety and stress [48]. Thus, the current findings may reflect life-stage-related dietary challenges rather than structural socioeconomic deprivation. Although women are typically reported to have higher diet quality than men [49], this relationship may vary depending on BMI and lifestyle factors. Some evidence suggests that when BMI is controlled for, low diet quality may be more strongly associated with higher BMI among women [49]. It is therefore plausible that the women in this cluster maintained a normal BMI but were near the upper threshold of normal weight, or that the high proportion of female respondents influenced the clustering outcome. Living in rural areas and larger households may also contribute to dietary limitations, including restricted food access, less dietary variety, and a greater reliance on calorie-dense foods [50]. Furthermore, single individuals tend to consume more convenience and fast food compared with married adults, who generally eat more legumes, fish, and nuts [51]. Taken together, this cluster likely represents younger, transitional individuals—predominantly female students—whose lower diet quality may stem from lifestyle constraints, social independence, and transitional living circumstances, rather than established socioeconomic hardship.

The second cluster included men, aged over 50, unemployed or retired, with less than secondary education, living alone, and showing low anxiety levels and high diet quality. At first glance, this finding contrasts with much of the literature, which generally associates unemployment and lower education with both increased anxiety and poorer diet [52]. Therefore, we explicitly interpret this cluster as an atypical and exploratory finding. Dietary quality is known to correlate inversely with anxiety and psychological distress [15]. Adherence to healthy dietary patterns—such as the Mediterranean or plant-based diet—has been associated with reduced stress and anxiety levels in several studies [53]. However, these associations are not universally confirmed; for example, in a meta-analysis by Paris et al. (2024), dietary interventions improved depressive but not anxiety symptoms among individuals with metabolic conditions [54]. This variability highlights that the diet–anxiety relationship may differ by age, health status, or psychological resilience. In the current sample, the coexistence of high diet quality with unemployment and low education may indicate a subgroup of financially stable or socially supported older adults, possibly retired individuals with established dietary routines. Research on older populations shows that individuals with healthy diets often report lower psychological stress, regardless of education level, and benefit from protective factors such as social activity [55]. However, this interpretation remains speculative in the absence of direct SES or social support measures. The third cluster comprised adults aged 23–50, with higher education, employed, overweight, in relationships, living in small urban households, and reporting moderate to high anxiety levels and moderate diet quality. Most studies associate higher anxiety with poorer diet quality [15], yet in this group, diet quality appeared better than expected. This may suggest a nuanced relationship in which anxiety does not uniformly predict unhealthy eating. One possible explanation is that health-related anxiety may encourage individuals to adopt healthier eating behaviors. In other words, moderate levels of anxiety might serve as a motivational factor for dietary improvement, especially among educated, health-aware adults. Evidence indicates that adherence to a Mediterranean diet is associated with reduced depressive symptoms, though findings for anxiety remain inconsistent [54,56]. Similarly, recent research on clustering of health behaviors suggests that individuals often exhibit mixed patterns—such as maintaining a relatively healthy diet while experiencing psychological distress [57]. The presence of overweight individuals in this cluster may reflect a population engaged in dietary improvement or weight-management efforts, which could explain the moderate (rather than low) diet quality observed. Indeed, longitudinal data show that overweight adults tend to report gradual improvements in dietary quality over time, particularly when motivated by health concerns [58]. The co-occurrence of employment and moderate anxiety might also be linked to occupational stress—an external factor not directly captured in our dataset. Cluster III may represent a transitional group: educated, working adults with moderate anxiety who are aware of their health risks and in the process of dietary change. The cross-sectional nature of the study, however, precludes determining the causal direction between anxiety and diet quality. Across all clusters, the interpretation of anxiety–diet relationships must be considered exploratory due to several limitations: absence of direct SES indicators, self-reported measures, and cross-sectional design. The current findings nonetheless provide a valuable basis for generating hypotheses about the complex interplay between sociodemographic factors, psychological well-being, and dietary habits. Considering the present findings and those of other authors, it can be concluded that the issue of anxiety and low-quality diets is widespread. The topic of diet and anxiety is a relatively new research area; therefore, further, more detailed studies are needed, both from a nutritional and psychological perspective, to determine whether diet affects anxiety levels or whether anxiety itself defines diet quality and influences the choice of specific products.

The discussion provides a broad overview of patterns observed across all respondents; however, the interpretation should mainly focus on the most represented demographic group—young female participants—whose responses substantially shaped the overall results. Given the cross-sectional design of this study, the observed associations between anxiety and diet quality should be interpreted with caution. These findings indicate co-occurrence rather than causation, as other unmeasured factors (e.g., alcohol consumption, smoking, lack of physical activity, or sleep disturbances) may confound or mediate this relationship. Due to the cross-sectional nature of this study, the direction of the relationship between anxiety and diet quality cannot be determined. Thus, it is not possible to conclude whether anxiety contributes to poorer dietary habits or whether unhealthy eating patterns exacerbate anxiety symptoms. The observed relationship should therefore be interpreted as correlational rather than causal.

### Strengths and Limitations of the Study

The data used in this study were collected from the Polish population; therefore, they cannot be generalized to other populations, especially those that differ culturally. The KomPAN questionnaire was used in the study, which has been validated among individuals up to 65 years of age, while this study also included participants over 65. Moreover, significantly more women than men took part in the study, which may have affected the reliability of the results. Most respondents were between 18 and 22 years old, which most likely resulted from the questionnaire being distributed via social media. Although the study included adult participants from across Poland, the sample was not representative of the national adult population. Women and younger adults, particularly those aged 18–25 years, were substantially overrepresented. The study sample was predominantly female, which is a common feature of online surveys, as women are generally more willing to share personal information, particularly thoughts and emotions [59]. (This gender imbalance may limit the generalisability of the findings. This demographic imbalance may limit the generalizability of the results, which should therefore be interpreted primarily in the context of young Polish adults, especially females. Future research using stratified or nationally representative sampling is needed to confirm whether the observed associations between anxiety and diet quality hold across other demographic groups. Participants recruited through online networks may differ from the general adult population in factors such as education, socioeconomic status, or health awareness. Another limitation of the study is the use of this newly developed diet quality index (“Road to Health”), which has not yet been formally validated. Although its construction was informed by national and international dietary guidelines as well as elements of existing validated tools, the lack of psychometric evaluation limits the interpretability of the results. The very low proportion of respondents classified in the “high diet quality” category may result from overly stringent criteria or insufficient sensitivity of the tool. The cross-sectional nature of the study precludes causal or temporal inferences; therefore, the observed associations should be interpreted as correlations only. Moreover, the interpretation of the identified clusters should be regarded as exploratory, as some explanations are speculative and based on unmeasured contextual factors. A key limitation of the present study is the lack of direct socioeconomic indicators, such as per capita or household income. Socioeconomic status is an important determinant of dietary patterns and mental health outcomes, and its omission may have influenced the observed associations between anxiety levels, diet quality, and eating behaviors. Although education level was included as a proxy for socioeconomic position, it does not fully capture the complexity of socioeconomic factors. Future research should therefore incorporate objective measures of socioeconomic status, including income or household expenditure, to more precisely evaluate their moderating role in the relationship between anxiety and dietary habits.

The study addressed issues related to anxiety, and some respondents may have discontinued participation due to reluctance to share such information or may have provided insincere responses. The paper raises an important current social issue—anxiety. The study confirmed the significance of this problem, indicating a high level of anxiety in society.

## 5. Conclusions

This study quantitatively assessed the association between anxiety levels and diet quality among Polish adults, revealing that nearly half of the respondents experienced high anxiety (48.29%) and that poor diet quality was reported by the majority (64.58%). Statistical analyses showed that higher levels of anxiety were significantly associated with poorer diet quality, with the strongest effects observed in the youngest age group (18–22 years).These results suggest that anxiety is related to diet quality, particularly among young adults. Several methodological limitations should be acknowledged. The study relied on self-reported measures, which may introduce reporting bias. The sample was not fully balanced across demographic groups, limiting generalizability. Additionally, the questionnaire was self-developed and not externally validated, which could affect the reliability of the diet quality and anxiety assessments. Future studies should employ longitudinal and experimental designs to clarify the causal direction between anxiety and dietary behaviors. Incorporating objective dietary assessments (e.g., dietary recalls or biomarkers), socioeconomic indicators (income, education, employment status), and biochemical as well as psychological measures could help better explain the underlying mechanisms linking anxiety and nutrition. Despite these limitations, the current findings provide robust empirical evidence of a statistically significant relationship between anxiety and diet quality. This underscores the potential value of integrating psychological and nutritional perspectives in future mental health and public health research, especially among young adults.

## Figures and Tables

**Table 1:** Study sample characteristics.

Variables	*N* = 1841	(%)
Gender		
Female	1693	91.96
Male	148	8.04
Age		
18–22 years old	1227	66.65
23–25 years old	279	15.15
26–50 years old	257	13.96
>50 years old	78	4.24
Body Mass Index		
Underweight	213	11.57
Normal weight	1053	57.20
Overweight	575	31.24
Place of residence		
Village	597	32.43
City < 100,000 residents	559	30.37
City ≥ 100,000 residents	685	37.21
Educational level		
Education lower than secondary	139	7.55
Secondary education	1162	63.12
Higher education	540	29.33
Employment status		
Student	1242	67.46
Unemployed	113	6.14
Working professionally	444	24.12
Pensioner	42	2.28
Marital status		
Single	962	52.25
In a relationship	879	47.74
Household structure		
Household of one	181	9.83
Household of two	408	22.16
Household of three	352	19.12
Household of four and more	900	48.89

**Table 2:** Distributions of anxiety levels and diet quality among study participants.

Variables	*N* = 1841	(%)
Anxiety level		
Low	238	12.93
Medium	714	38.78
High	889	48.29
Diet quality		
Low	1189	64.58
Medium	638	34.66
High	14	0.76

**Table 3:** Multivariable logistic regression analysis of factors associated with high anxiety level (H_A).

Variables	*p*	aOR	95% CI	
			Lower Limit	Upper Limit
**High anxiety level (H_A)**				
W	<0.001	2.926	1.999	4.282
18–22	<0.001	1.614	1.327	1.964
23–25	0.344	1.131	0.876	1.459
>50	<0.001	0.263	0.151	0.459
U	0.005	1.505	1.128	2.008
O	0.737	1.034	0.849	1.260
V	0.385	1.090	0.897	1.325
C < 100	0.924	0.990	0.812	1.208
L_SE	0.389	1.164	0.824	1.645
HE	0.002	0.731	0.597	0.894
ST	<0.001	1.621	1.330	1.975
UE	0.389	1.182	0.808	1.730
P	0.002	0.327	0.160	0.669
S	0.672	0.961	0.800	1.154
1H	0.826	0.966	0.711	1.314
2H	0.177	0.859	0.689	1.071
3H	0.815	0.973	0.771	1.227

Reference categories: Gender = Men; Age = 26–50 years; BMI = Normal weight; Place of residence = City ≥ 100,000; Education = Secondary; Employment status = Working professionally; Marital status = In a relationship; Household = Household of four and more; W—women; 18–22—age in years; 23–25—age in years; >50—age in years; U—underweight; O—overweight; V—village; C < 100—city with up to 100,000 inhabitants; L_SE—education lower than secondary; HE—higher education; ST—students; UE—unemployed; P—pensioners; S—single; 1H—household of one; 2H—household of two; 3H—household of three.

**Table 4:** Multivariable logistic regression analysis of factors associated with low diet quality (L_Q).

Variables	*p*	aOR	95% CI	
			Lower Limit	Upper Limit
**Low diet quality (L_Q)**				
W	0.175	1.268	0.900	1.787
18–22	<0.001	1.810	1.482	2.211
23–25	0.028	0.747	0.576	0.969
>50	0.566	0.872	0.547	1.391
U	0.002	1.663	1.204	2.298
O	0.039	1.246	1.011	1.536
V	<0.001	1.466	1.189	1.807
C < 100	0.398	1.094	0.888	1.348
L_SE	0.002	1.916	1.275	2.879
HE	<0.001	0.513	0.417	0.630
ST	0.005	1.333	1.089	1.630
UE	0.043	1.556	1.013	2.389
P	0.049	0.540	0.293	0.997
S	0.946	1.007	0.831	1.219
1H	0.023	0.698	0.511	0.953
2H	0.002	0.700	0.559	0.877
3H	0.591	0.936	0.735	1.192

Reference categories: Gender = M; Age = 26–50 years; BMI = Normal weight; Place of residence = City ≥ 100,000; Education = Secondary; Employment status = Working professionally; Marital status = In a relationship; Household = Household of four and more; W—women; 18–22—age in years; 23–25—age in years; >50—age in years; U—underweight; O—overweight; V—village; C < 100—city with up to 100,000 inhabitants; L_SE—education lower than secondary; HE—higher education; ST—students; UE—unemployed; P—pensioners; S—single; 1H—household of one; 2H—household of two; 3H—household of three.

## Data Availability

The original contributions presented in this study are included in the article. Further inquiries can be directed to the corresponding author.
